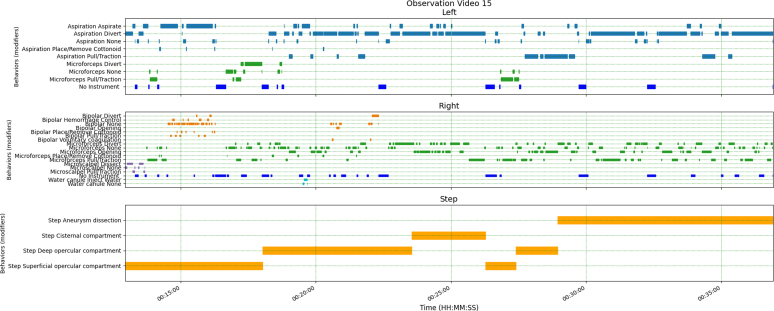# Revisiting sylvian fissure dissection - A preliminary investigation into surgical process modelling for evaluating surgical proficiency

**DOI:** 10.1016/j.bas.2025.104284

**Published:** 2025-05-21

**Authors:** Félix Buyck, Jef Vandemeulebroucke, Ine Dirks, Jan Frederick Cornelius, Johnny Duerinck, Sebastien Froelich, Henry Schroeder, Wietse Geens, Frederick Van Gestel, Michaël Bruneau

**Affiliations:** a Department of Neurosurgery, Universitair Ziekenhuis Brussel (UZ Brussel), 1090, Brussels, Belgium; b Vrije Universiteit Brussel (VUB), Research group Center for Neurosciences (C4N-NEUR), 1090, Brussels, Belgium; c Vrije Universiteit Brussel (VUB), Department of Electronics and Informatics (ETRO), 1050, Brussels, Belgium; d Department of Radiology, Universitair Ziekenhuis Brussel (UZ Brussel), 1090, Brussels, Belgium; e Imec, 3001, Leuven, Belgium; f Department of Neurosurgery, Medical Faculty, Heinrich-Heine-University, 40225 Düsseldorf, Germany; g Department of Neurosurgery, Lariboisière Hospital, 75010, Paris, France; h Department of Neurosurgery, Medicine Greifswald University, 17489, Greifswald, Germany

**Keywords:** Surgical process modelling, Skill assessment, Surgical video analysis, Sylvian fissure dissection, Proficiency

## Abstract

**Introduction:**

Understanding the factors that contribute to efficient surgical behaviour and the prevention of technical errors poses a significant challenge in neurosurgery. Current training curricula lack proficiency-centred training and objective tools to assess surgical performance, leading to considerable variability in surgical competencies and practices among neurosurgeons. This study aims to evaluate the determinants of proficient surgical behaviour exhibited by expert surgeons, with the goal of establishing a set of surgical performance metrics serving as a foundation for objective assessment and benchmarking of surgical performance.

**Material and methods:**

Eight aneurysm clipping cases by three senior neurosurgeons were recorded via a surgical microscope. Surgeons' actions, workflow parameters, and adverse events during Sylvian fissure dissection were catalogued into Surgical Process Models (SPMs). Performance metrics were extracted, compared, and analysed using clustering analysis to assess proficiency differences.

**Results:**

23 parameters were identified as potential metrics of surgical proficiency. Proficient surgeons exhibited predominant bimanual activity, optimal non-dominant hand use, a limited tool repertoire, minimal instrument changes, and efficient microscope use with minimal adjustments. Despite varying instrument and microscope usage, practitioners achieved consistent outcomes across metrics, indicating similar surgical proficiency.

**Discussion and conclusion:**

Findings illustrate that performance metrics derived from surgical video analysis can reliably contribute to the assessment of surgical skills. SPMs offer a structured understanding of the factors that contribute to surgical proficiency. This approach provides an optimal framework for objective assessment of performance metrics, demonstrating potential for automated and objective analysis of surgical performance.

## Highlights

• This study analysed key factors of proficient surgical behaviour in surgical recordings of expert surgeons, utilizing a surgical process model.
• 23 parameters were identified as potential metric of surgical proficiency.
• Findings indicate that performance metrics obtained from surgical video analysis can serve as a reliable means for evaluating surgical skills.
• SPM offers an objective framework for assessing performance metrics, underscoring its capacity for automated analysis of surgical proficiency.

## 1 Introduction

Training in the field of neurosurgery is a traditional system based on a peer-to-peer transmission of knowledge, with practical experience at its core. Surgical training is classically done by reproducing the surgical technique of senior neurosurgeons (Angelo et al., 2015; Gallagher, 2012). However, surgical techniques and used instruments are not only operator-dependent but may also vary from one centre to another. Consequently, there is a considerable heterogeneity in the skills that neurosurgeons are taught. Another pitfall is the absence of proficiency-centred training and feedback (Gallagher, 2018). Furthermore, there is also considerable variability on the amount of cases in which young neurosurgeon are involve and their level of autonomy. Ultimately this cumulates in a significant heterogeneity in surgical competencies, which may affect the quality of the surgical care (Angelo et al., 2015).

In order to optimize surgical training in terms of reliability and efficiency, it is paramount to create a tool that allows objective assessment of surgical performance.

Videos and images are considerably richer in information and more objective as opposed to skill grading models which rely on recall of subjective observations of an evaluator (Buyck et al., 2023, Knopf et al., 2020; Sarkiss et al., 2016). Moreover, these evaluations often take place in the post-operative setting, often leading to a certain loss of information. Thus, more emphasis should be placed on the intervention itself, which is often recorded during microscope and endoscope assisted interventions.

Surgical process modelling (SPM) is a technique that attempts to describe a surgical intervention in a comprehensive manner using a standardised and structured terminology. SPM essentially involves the development of a model that provides a structured representation of surgical actions, milestones, and adverse events in a chronological manner. This structured representation aims to delineate the dynamics and proficiency of the performance during the surgical procedure (Lalys and Jannin, 2014).

The application of such models provides a valuable framework for evaluating the effectiveness of surgical behaviour or the assessment of any facilitating and constraining factors that influence the quality of a procedure. In the field of neurosurgery, this framework has seen implementation in various types of procedures such as lumbar (Riffaud et al., 2010) and cervical intervertebral disc surgery (Forestier et al., 2013) or pituitary surgery (Laurent Riffaud, 2012) as well as the peri-operative environment (operating theatre) (Morineau et al., 2015).

Our study seeks to implement surgical process models specifically for the dissection of the Sylvian fissure, a classic approach to a deep target in neurosurgery. The primary objective is to assess the determinants of proficient surgical skills exhibited by expert surgeons during sylvian fissure dissection. Through analysis of variances in surgical practice among centres and various levels of experience, we aim to establish a set of surgical performance metrics that will serve as a base for objective assessment and benchmarking of surgical performance. These metrics will not only provide a reference to distinguish the level of experience but also instigate deliberate practice through objective, transparent and quantitative milestones to obtain a predefined performance benchmark (Gallagher et al., 2022). Furthermore, this study seeks to explore the feasibility of automating the recognition of surgical performance metrics.

## 2 Methods and materials

### 2.1 Study design and patient inclusion

This pilot study was designed as a retrospective observational study of patients undergoing cranial surgery wherein a sylvian fissure dissection was performed with use of a surgical microscope. The protocol and design were approved by the local ethics committee of UZ Brussel. Patients were recruited in three different neurosurgical centres in Europe in the period between September 2021 and May 2022. Prior to participation, all patients signed an informed consent.

Cases related to subarachnoid haemorrhages or underlying pathology inducing mass effect leading to significant distortion of the cerebral anatomy were not eligible for this study. Only patients whose procedures were recorded using a surgical microscope were included to ensure consistent image quality.

Surgical recordings had to be uninterrupted from the beginning of the sylvian fissure dissection until the end of the procedure. Only original clips were approved for inclusion, any edited footage (trimming, zooming, annotation, etc.) was excluded from this study. No restrictions were imposed on the use of the microscope (zooming, angulation, etc.).

### 2.2 Data acquisition

Intra-operative recordings (60fps, 1080p) were captured during the procedure using a surgical microscope and subsequently collected post-operatively.

The beginning of analysis started at dura opening. Given anatomical variations of the sylvian fissure as well as the different indications for surgery, a definite demarcation to the ending of the sylvian fissure dissection could not be defined. Therefore the recording was terminated at the moment the surgical target (i.e. the aneurysm or the tumour)had been achieved. Throughout the course of the intervention surgical recordings could not be uninterrupted.

### 2.3 Data analysis

Data was manually annotated by one evaluator (F.B.) by the use of BORIS event-logging software (Friard and Gamba, 2016). The initiation of the sylvian fissure dissection was marked by the first surgical action on the Sylvian fissure. On the other hand, the ending of the analysis was marked by the surgical action initiating the clipping phase.

Prior to analysing the data, an ethogram was developed which encompassed an inventory of all the different objects and behaviours of interest that would be annotated (Supplementary data). The annotation of each surgical event was constituted by (1) subject (2) surgical event (3) behavioural state (4) modifier.

A total of 10 different surgical instruments were analysed. When surgical instruments were not visibly present, surgical occurrences were designated as "no instrument," indicating the absence of tools in either the left or right hand (Fig. 1). The instruments were deemed to be in an idle surgical state when they exhibited no active surgical behaviour, the behaviour of the tools was then labelled as "none." This also included the time ranging between the appearance of a surgical tool to the actual surgical action. The interval during the idle state denoted the period wherein a surgeon engaged in decision-making and motor-planning activities (D'Angelo et al., 2015; Oropesa et al., 2011).

**Fig. 1:**
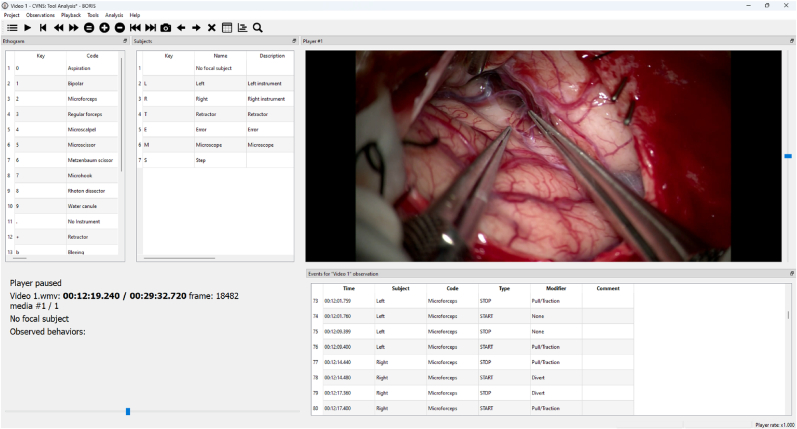
Illustration of the annotation software. The top left “Ethogram” pane displays the various parameters that compose a surgical event (surgical tools, microscope usage, retractor usage, haemostatic usage, errors). Top middle “Subject” pane displays the various actors to which surgical events are linked (left hand, right hand, retractor, microscope and error).

The investigation also examined the utilization of the microscope in terms of its duration of use, the frequency of adjustments in focus/zoom and positional modifications. Brain retraction was assessed by means of the application of a retractor. With regards to adverse events, the frequency of haemorrhages, cortical lesions and pial retraction was assessed respectively. Within this context the control of adverse events was measured in terms of haemostatic agent (i.e. haemostatic mesh or liquid) application as well as the frequency and relative duration of use of the bipolar.

Upon the conclusion of the labelling process, a tabulated summary was generated of all labelled surgical events and single observations. To mitigate potential bias arising from procedures with extended operating times, all parameters were standardized and expressed as frequencies per minute (/min). This normalization accounts for the inherent variability introduced by procedures of varying durations, given that longer surgeries inherently encompass a greater number of surgical actions and adverse events.

In the final stage, a comparison of the Surgical Process Models (SPMs) was conducted in Python 3.10 (Python Software Foundation) using hierarchical clustering analysis with a Dynamic Time Warping (DTW) algorithm, as described by Forestier et al. (2012) (Supplementary data). The analysis allowed to assess the degree of similarity in surgical activities within the respective cases. Consequently, surgical cases demonstrating high resemblance in workflow or surgical behaviour would be clustered together or separately depending of the degree of dissimilarity. All calculations were performed using scipy 1.10.1 and numpy 1.24.3.

## 3 Results

### 3.1 Intervention and surgeons

15 cranial interventions involving the dissection of the sylvian fissure were recorded in three different surgical centres in Europe. Seven from originated from centre A, four from centre B, four from centre C. Each of the recordings included the sylvian fissure dissection as part of the procedure. Interventions were performed by three different senior surgeons, with assistance of a resident (=post-graduate) neurosurgeon. Interventions involved eight cases of sylvian aneurysm clipping and seven cases of tumour resection.

Four recordings were discarded from analysis because the footage was fragmented, thus not containing the totality of the intervention. Two cases was discarded because the intervention was associated with subarachnoid haemorrhage. One case was discarded because the underlying pathology induced a mass effect leading to significant distortion of the cerebral anatomy. The remaining recordings exclusively consisted of cases of aneurysm clipping. Five interventions were performed in centre A, two interventions were performed in centre B and one in centre C. 50 % of the cases involved a blunt dissection of the sylvian fissure, 25 % a sharp dissection and 25 % involved a hybrid blunt/sharp approach.

The dissection of the sylvian fissure was divided into the following phases (Tayebi et al., 2019):

1. Dissection of superficial opercular compartment
2. Dissection of deep opercular compartment
3. Dissection of cisternal compartment

A total of 7.241 surgical events were labelled, accounting for 5h 44min 3sec of annotated surgical footage. In terms of the labelling procedure, 1 h of manual analysis could yield up to 15–20min of the operational duration pertaining to (1) left-hand gestures, (2) microscope manipulations, (3) errors. In contrast, the same duration of manual analysis was able to yield up to 15–20min of the operational duration relating to right-hand gestures, which were more complex to analyse. The results of these annotations are summarised in (Supplementary Tables 2-4).

### 3.2 Duration of intervention

The median duration of the intervention starting from the opening of the arachnoid membrane until first attempt of clipping was 27min 15sec (Q1: 23min 23sec, Q3: 47min 27sec). Concerning the distinct stages of the intervention, surgeons allocated the greatest amount of time to the dissection of the aneurysm. Notably, this phase exhibited the greatest variability in time-expenditure. The specific durations for each stage of the procedure are summarised in Fig. 2 and Supplementary Table 1.

**Fig. 2:**
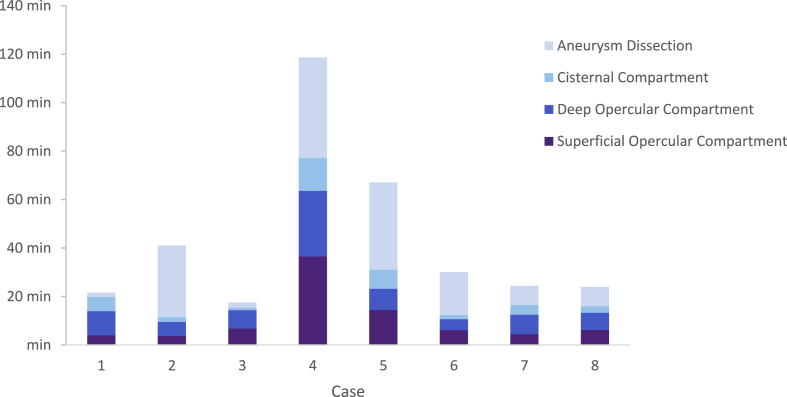
Proportional representation of surgical activity per phase per intervention.

### 3.3 Surgical efficiency

The average frequency of tool changes observed was 0.69/min (SD: 0.19). Instrument switches performed by the right hand were considerably more frequent as opposed to those performed by the left hand (p < 0.001). Examination of surgical events indicated an overall average frequency of 4.86 surgical actions per minute. No significant differences were observed in the frequencies of surgical actions performed by the left and right hand throughout the entire procedure, including specific surgical phases.

On average, the percentage of surgical inactivity displayed by the different surgeons accounted for 11.43 % (SD: 5.06) of the total surgical procedure. The average time from an idle state to active surgical conduct was 4.32sec (SD: 5.54). There was no statistically significant difference between the idle time displayed by the left and right hand. With regards to active instrument application, surgeons displayed a higher overall percentage of bi-manual surgical activity 65.32 % (SD: 4.40) as opposed to single handed tool application 34.68 % (SD: 4.40). In turn, single handed activity was predominantly left-handed. Components of the surgical activity are summarised in Fig. 3.

**Fig. 3:**
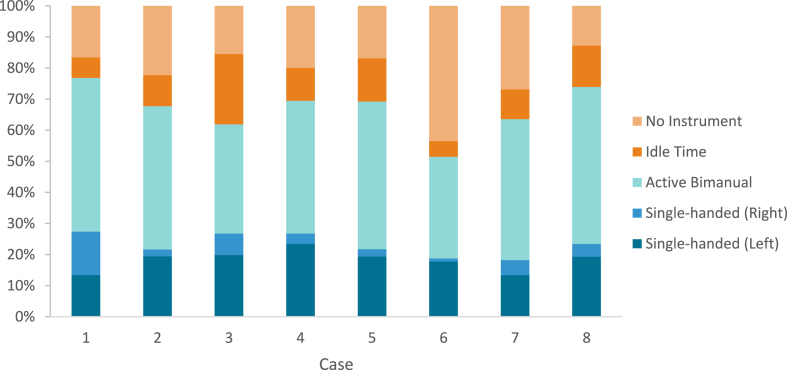
Proportional representation of active (bi-manual or single-handed), idle and inactive surgical time per intervention.

### 3.4 Tool application

The average number of instruments used across the different surgical procedures was 10 (SD: 0.66). The various instruments and their respective use percentage are summarised in Supplementary Table 4. The use of retractors was restricted to two of the eight cases.

### 3.5 Adverse events

The occurrence of adverse events was limited (M:0.16/min, SD: 0.10), with only a few occurrences of minor haemorrhages throughout an entire intervention (M: 4.63, SD: 2.14) and in one case a small bleeding from larger venous vessel. Surgeons exhibited proficient control of these events, necessitating an average of one coagulation per 10min of intervention (SD: 0.13), with each coagulation lasting on average 2.92sec (SD: 3.08).

Moreover, the use of haemostatic agents was rare. There was only one incident of a cortical lesion, related to a thermal injury as a byproduct of cauterisation.

### 3.6 Microscope

Microscope adjustments varied according to the difficulty of the procedure. On average, the frequency of modifications amounted to 0.87/min (SD: 0.22) and mostly involved changes in focus or zoom (M: 0.64/min, SD: 0.19) – of which on average 7.25 % (SD: 16.21) was executed by the assisting surgeon – rather than changes in position (M: 0.23/min, SD: 0.09). On average, microscope adjustments made by the operating surgeon accounted for 5.95 % (SD: 1.99) of the total operating time.

### 3.7 Hierarchical clustering analysis

The clustering analysis identified two clusters, C1 and C2. The former (C1), contained all five cases performed at site A as well as the single case performed at site C, performed by surgeon N° 1 and N° 3 respectively. The latter (C2), contained all two cases performed at site B, performed by surgeon N°2. The results of this hierarchical clustering analysis are displayed in Fig. 4. In this figure, the Y-axis represents the DTW dissimilarity index, expressed as the distance between the sequences of events. In essence, interventions displaying high similarity in their surgical process models are iteratively connected in pairs (displayed as inverse brackets) to form clusters. The height of the brackets which form a cluster is proportional to the dynamic time warp (DTW) index, illustrating the degree of dissimilarity between interventions. Clustered cases typically present a smaller DTW index, which corresponds to a greater similarity between the SPMs of surgical operators.

**Fig. 4:**
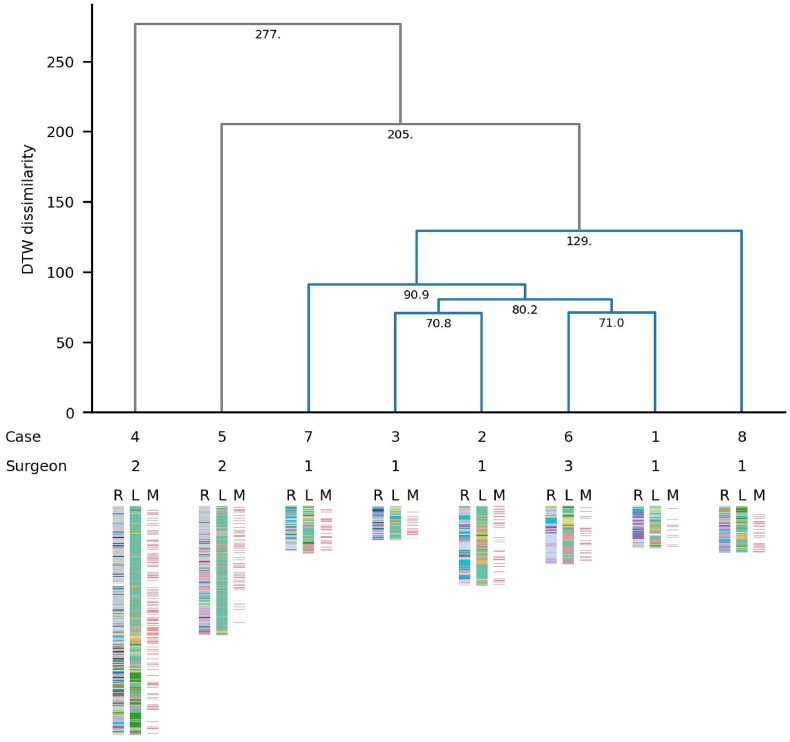
Hierarchical clustering of surgical performances. Each case of a Sylvian fissure dissection is represented by a Surgical Process Model (SPM), which is a structured depiction of the surgical actions performed during the intervention. Each SPM is divided into three vertical columns (index-plots): R (right hand), L (left hand), and M (microscope). Each of these columns comprises coloured bars that represent individual surgical actions performed by the corresponding subject in chronological order. Each colours corresponds to a specific triplet of modifiers {subject; tool; activity}. The height of each coloured bar indicates the duration of that surgical action. Two primary clusters are identified: cluster 1 (blue) and cluster 2 (grey).

## 4 Discussion

The SPM approach demonstrated the potential to elucidate the intricate nuances of surgeons' operative behaviour. Ultimately, these insights may contribute to a better understanding of the factors influencing efficient surgical performance or potentially triggering specific adverse events.

The formal categorisation of the surgical actions according to a pre-defined structure based and through analysis of surgical recordings not only to facilitate comparison between surgeons but also to expand the possibilities of skill assessment beyond the generic performance criteria or amalgams of different parameters used in traditional evaluation models (Doyle et al., 2007).

### 4.1 Duration of surgery

The duration of the various interventions was widely varying despite the similar levels of experience of the surgeons. This substantiates the notion that the overall duration of the surgical procedure inadequately predicts surgical proficiency (Forestier et al., 2013; Riffaud et al., 2010). In contrast, the surgeon's workflow, efficiency in active use of instruments as well as surgical behaviour have showed to offer more comprehensive assessment of technical competence.

Concerning the distinct surgical phases of sylvian fissure dissection, surgeons exhibited efficient advancement through different compartments, minimizing the need for retroactive adjustments in prior and more superficial areas. This finding substantiates the concept of “back-tracking”, which appears to be proportionally more frequent amongst less experienced surgeons (Morineau et al., 2015). It is important to note however, that in this case surgeons did demonstrate a certain degree of retroactive operating, but this stemmed from the utilization of the inside-to-outside technique (Cohen- and Gadol, 2020).

The intricacy of the intervention is intricately tied to the anatomy and location of the aneurysm. Omission of the aneurysmal dissection stage leads to a significant reduction in variability across the preceding three phases. The substantial resemblance in surgical efficiency observed in the stages preceding aneurysm dissection suggests an automation of both technical and cognitive skills, underscoring a notable and consistent level of proficiency among these experienced surgeons.

Beyond the evaluation of surgical activity, this study also considered periods of inactivity within the assessment of proficiency. While there is no established threshold for the duration of idle time in relation to the surgical experience, studies across different surgical disciplines have demonstrated the significance importance of this metric in evaluating and distinguishing surgical proficiency (D'Angelo et al., 2015; Deepika et al., 2022, 2023; Mackenzie et al., 2021)–(D'Angelo et al., 2015; Deepika et al., 2022, 2023; Mackenzie et al., 2021). Nevertheless, the significance of variations in idle time across different levels of surgical expertise and the threshold for proficient surgical activity have yet to be formally assessed.

### 4.2 Surgical efficiency

From a technical perspective, surgeons demonstrated infrequent occurrences of tool changes as well as a limited use of different surgical tools, which contributed to limited interruptions of the workflow. Previous research from Riffeaud et al. reported mean frequencies of gestures ranging between 1.4 and 1.68/min and 0.28–0.30/min in lumbar disc herniation procedures and pituitary tumour surgery. Their result showed that expert surgeons were more economical with their movements as opposed to junior surgeons, yet displayed a higher number of surgical actions with their non-dominant hand (Laurent Riffaud, 2012; Riffaud et al., 2010).

In contrast to Riffeaud et al.'s results, our findings demonstrated a higher similarity in the frequency of surgical gestures between the dominant (2.81/min, SD: 1.44) and non-dominant (2.05/min, SD: 0.75) hands, suggesting surgeons optimized their surgical efficiency by increasing the activity of their non-dominant hand. This discrepancy might be partially related to the type of intervention.

Although surgeons predominantly engaged in bimanual activity, the percentage of single-handed activity exceeded expectations (Mean: 47.02 %, SD: 15.14). However, this proportion may be skewed due to time invested in the installation of a retractor and microscope adjustments, both executed by the dominant hand only. When taking into consideration of the factors within the surgical activity, the distribution of single-handed and bimanual activity significantly shifts in favour of bimanual activity (approximately 63.5 %).

Surgeons displayed minimal repositioning and focus/zoom adjustments of the microscope throughout the intervention. This finding concurs with earlier reports indicating experienced neurosurgeons exhibit a more economical use of microscope adjustments compared to intermediate or junior surgeons. Notably, the microscope applications observed in our study were significantly lower than those reported in the literature (0.87/min vs 1.80/min).

In summary, surgeons demonstrated swift and proficient surgical behaviour, potentiated by pre-dominant bi-manual activity, optimal utilization of the non-dominant hand, limited tool repertoire and occurrences of instrument changes as well as efficient use of microscope.

### 4.3 Surgical consistency 4.3

Despite subtle differences in surgical performance measurements, practitioners consistently demonstrated uniform outcomes across various metrics as a whole, thus conveying a sense of common degree of surgical proficiency. This pattern was even more notably present in the examination of the cases conducted by the same surgeon (operator 1). This can be explained in part by a self-loop feedback and personal strategies to improve its own technical skills, which ultimately improve surgical proficiency and surgical consistency. This was apparent within the hierarchical clustering analysis, which accurately distinguished the different surgical operators based on patterns surgical workflow and behaviour. While not yet systematically studied, we propose that the variability of surgical parameters across interventions may serve as valuable indicator of surgical experience.

### 4.4 Limitations

This study presents certain limitations.

Firstly, there was a significant class imbalance in the number of surgeons across the various surgical centres. Therefore, the comparison and interpretation of the assessed parameters were likely subject to bias, potentially leading to the omission of certain proficiency metrics, disproportionate weighting of their importance, or inaccurate representation of the true performance standards of individual surgeons. To address this limitation, we utilized relative frequencies rather than absolute values to enhance the descriptive analysis of the surgical process models.

Secondly, we could not assimilate any information regarding the complexity of the case which undoubtably impacts the surgical performance. The degree of complexity was thus based upon the visual cues, occurrence of adverse events and duration of the different surgical phases (mostly the duration of the aneurysm dissection).

Thirdly, the validation of our results was constrained owing to the fact that the data was annotated by only a single individual. This limitation could be mitigated by using collaborative annotation software that enables second-party validation and correction of annotations. Although we did not include a control group for performance comparison, incorporating multiple cases from different operators with similar experience levels would provide a more reliable analysis of performance metrics. However, within the scope of this pilot study, this was not feasible.

Finally, this pilot study did not include any cases performed by novices or neurosurgeons with intermediate experience. The establishment of a larger study population, including novice and intermediate surgeons, will be critical to discerning performance metric variations across different levels of experience and the establishment of performance benchmarks in subsequent research.

## 5 Conclusion

In conclusion, the surgical process modelling undertaken in this pilot study has proven effective in capturing the intricate nuances of surgeons' operative behaviour, thereby offering a more comprehensive understanding of the factors contributing to their overall proficiency as opposed to traditional evaluation models. The findings suggest that performance metrics derived from the analysis of surgical practices in recordings of microsurgical procedures can serve as reliable indicators for assessing surgical skills.

Furthermore, the standardized and structured reporting provided by these surgical process models creates a framework that could facilitate the automated comparison of performance metrics using computer-vision techniques. This potential for automation offers promising prospects for objective and automatic performance assessment, enabling the detection of adverse events and other critical metrics. In the future, these advancements in surgical performance assessment and guidance could substantially enhance neurosurgical training and the development of proficient skills among practitioners.

## Disclosures

The authors report no conflict of interest. This research did not receive any specific grant from funding agencies in the public, commercial, or not-for-profit sectors.

## Disclosures

The authors report no conflict of interest.

## Figures and Tables

**figs1:**
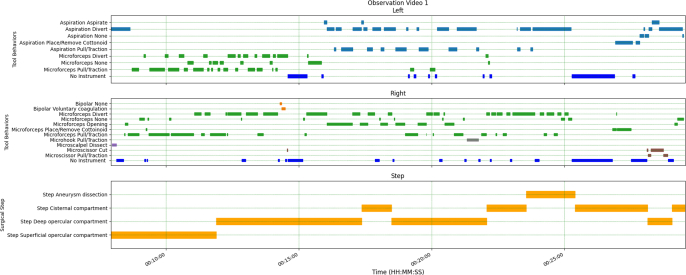


**figs2:**
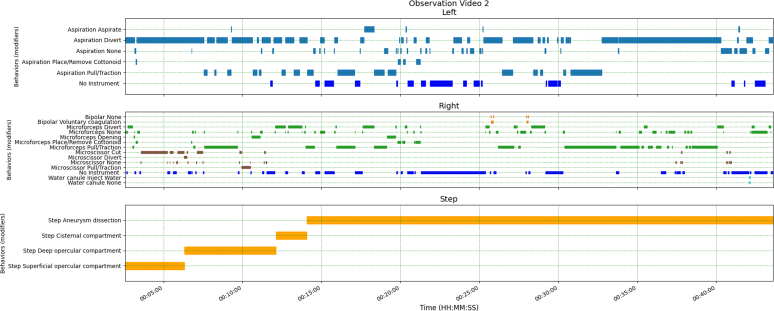


**figs3:**
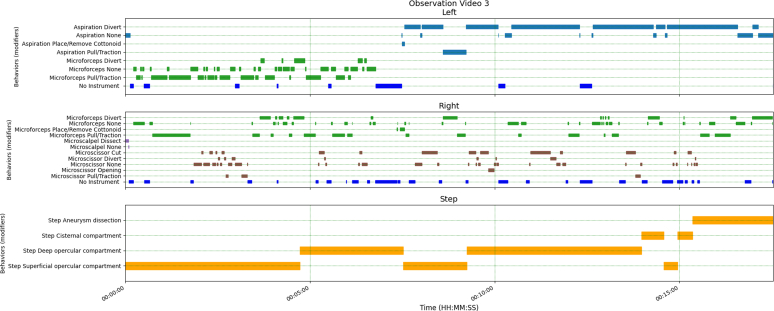


**figs4:**
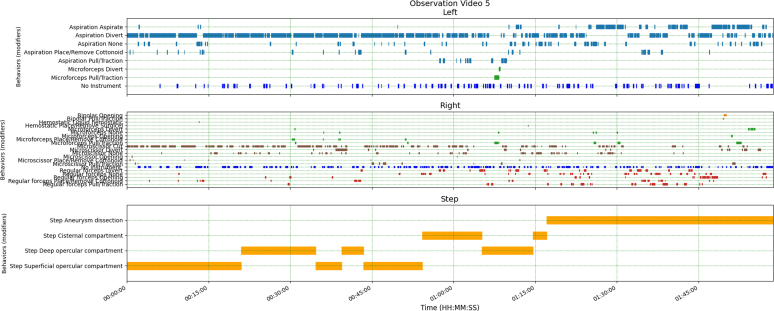


**figs5:**
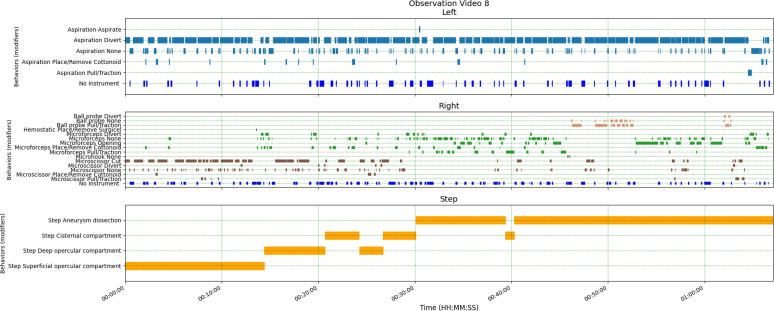


**figs6:**
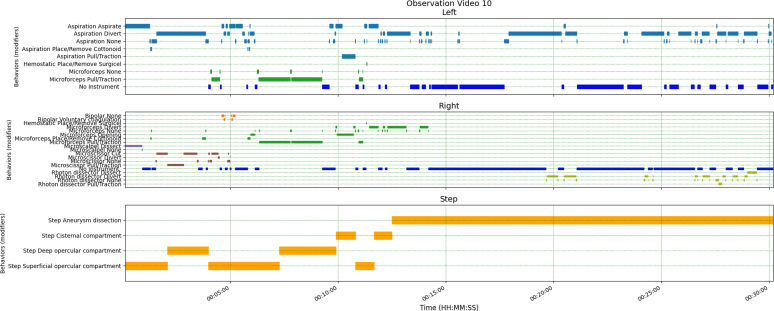


**figs7:**
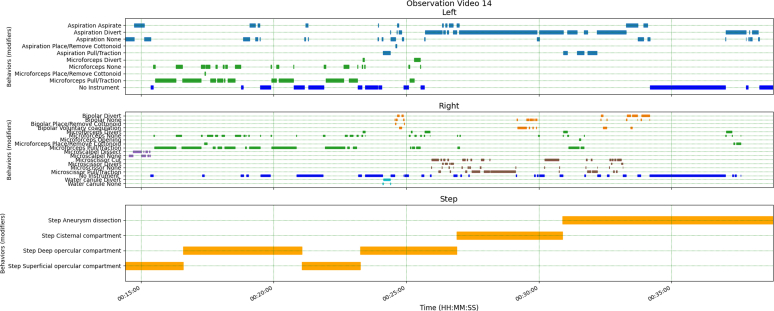


**figs8:**