# Supplementary material for: Revisiting sylvian fissure dissection - A preliminary investigation into surgical process modelling for evaluating surgical proficiency
Source: Brain Spine. 2025 May 21;5:104284. doi: 10.1016/j.bas.2025.104284 (PMC12171760; doi:10.1016/j.bas.2025.104284)
Supplement: Multimedia component 1 [file mmc1.docx]

| **TABLE 1 \| Ethogram** | | | |
| --- | --- | --- | --- |
| **Subject** | **Surgical event** | **Behaviour type** | **Modifier** |
| Left Hand  Right Hand | Aspiration | State event | None , Aspirate , Divert , Pull/Traction , Place/Remove Cottonoid |
|  | Bipolar | State event | None , Divert , Pull/Traction , Opening , Voluntary coagulation , Haemorrhage Control , Place/Remove Cottonoid |
|  | Microforceps | State event | None , Divert , Pull/Traction , Opening , Place/Remove Cottonoid |
|  | Regular forceps | State event | None , Divert , Pull/Traction , Opening , Place/Remove Cottonoid |
|  | Microscalpel | State event | None , Divert , Pull/Traction , Dissect |
|  | Microscissor | State event | None , Divert , Pull/Traction , Opening , Cut , Place/Remove Cottonoid |
|  | Metzenbaum scissor | State event | None , Divert , Pull/Traction , Opening , Cut |
|  | Microhook | State event | None , Divert , Pull/Traction , Place/Remove Cottonoid |
|  | Rhoton dissector | State event | None , Divert , Pull/Traction , Dissect , Place/Remove Cottonoid |
|  | Water cannula | State event | None , Inject Water (0), Divert , Pull/Traction , Place/Remove Cottonoid |
|  | Ball probe | State event | None , Divert , Pull/Traction , Place/Remove Cottonoid |
|  | No Instrument | State event |  |
|  | Haemostatic | Point event | Place/Remove Surgicel , Liquid haemostatic |
| Retractor | Retractor | Point event | Placement , Removal |
| Adverse event | Bleeding | Point event | Small vessel , Big vessel |
|  | Pial retraction | Point event |  |
|  | Cortical lesions | Point event | Tissue tearing , Pressure ischemia , Thermal injury |
| Microscope | Microscope adjustment | State event | Zoom/Focus , Change of position |
| Phase | Phase | State event | Superficial opercular compartment , Deep opercular compartment , Cisternal compartment , Aneurysm dissection |
