# Supplementary material for: Revisiting sylvian fissure dissection - A preliminary investigation into surgical process modelling for evaluating surgical proficiency
Source: Brain Spine. 2025 May 21;5:104284. doi: 10.1016/j.bas.2025.104284 (PMC12171760; doi:10.1016/j.bas.2025.104284)
Supplement: Multimedia component 2 [file mmc2.docx]

| **TABLE 2 \| Surgical Efficiency** | **Case 1** | **Case 2** | **Case 3** | **Case 4** | **Case 5** | **Case 6** | **Case 7** | **Case 8** | **Mean** | **Std dev.** |
| --- | --- | --- | --- | --- | --- | --- | --- | --- | --- | --- |
| **N° tool changes (total)** | 0.74/min | 0.44/min | 0.97/min | 0.59/min | 0.58/min | 0.50/min | 0.98/min | 0.75/min | 0.69/min | 0.19 |
| N° tool changes left hand | 0.37/min | 0.00/min | 0.00/min | 0.02/min | 0.00/min | 0.20/min | 0.33/min | 0.33/min | 0.16/min | 0.16 |
| N° tool changes right hand | 0.37/min | 0.44/min | 0.97/min | 0.57/min | 0.58/min | 0.30/min | 0.66/min | 0.42/min | 0.54/min | 0.20 |
| **N° surgical actions (total)** | 5.64/min | 2.90/min | 5.13/min | 3.84/min | 3.70/min | 2.76/min | 5.12/min | 9.81/min | 4.86/min | 2.12 |
| N° actions left hand | 2.82/min | 1.49/min | 1.77/min | 1.71/min | 1.42/min | 1.60/min | 1.88/min | 3.71/min | 2.05/min | 0.75 |
| N° actions right hand | 2.82/min | 1.41/min | 3.36/min | 2.13/min | 2.28/min | 1.16/min | 3.23/min | 6.09/min | 2.81/min | 1.44 |
| **N° surgical actions (surgical phase)** |  |  |  |  |  |  |  |  |  |  |
| Superficial opercular compartment | 5.81/min | 2.39/min | 6.44/min | 3.56/min | 1.53/min | 5.39/min | 3.19/min | 9.84/min | 4.77/min | 2.50 |
| Deep opercular compartment | 6.51/min | 3.97/min | 4.66/min | 0.33/min | 8.10/min | 2.44/min | 7.03/min | 10.33/min | 5.42/min | 3.01 |
| Cisternal compartment | 4.12/min | 4.11/min | 4.99/min | 3.85/min | 3.31/min | 4.04/min | 4.51/min | 11.37/min | 5.04/min | 2.44 |
| Aneurysm dissection | 5.43/min | 2.68/min | 2.75/min | 4.21/min | 3.55/min | 1.81/min | 4.54/min | 8.78/min | 4.22/min | 2.04 |
| **No instrument (total)** | 16.60% | 22.3% | 15.05% | 20% | 16.9% | 43.5% | 26.9% | 12.8% | 21.76% | 9.21 |
| **Idle time (total)** | 6.60% | 9.95% | 22.60% | 10.50% | 13.90% | 5.05% | 9.55% | 13.30% | 11.43% | 5.06 |
| Idle time left hand | 4.00% | 4.1% | 10.2% | 4.1% | 4.6% | 2.95% | 3.3% | 3.45% | 4.59% | 2.18 |
| Idle time right hand | 2.60% | 5.85% | 12.4% | 6.4% | 9.3% | 2.1% | 6.25% | 9.85% | 6.84% | 3.32 |
| **Active time (total)** | 76.80% | 67.75% | 61.90% | 69.50% | 69.20% | 51.45% | 63.55% | 73.90% | 66.76% | 7.37 |
| **Single-handed activity (active time)** | 35.64% | 31.95% | 43.2% | 38.48% | 31.43% | 36.46% | 28.65% | 31.64% | 34.68% | 4.40 |
| Left hand | 17.44% | 28.67% | 32.04% | 33.64% | 27.97% | 34.48% | 21.04% | 26.15% | 27.68% | 5.62 |
| Right hand | 18.20% | 3.28% | 11.16% | 4.84% | 3.46% | 1.98% | 7.61% | 5.49% | 7.00% | 5.02 |
| **Bi-manual activity (active time)** | 64.36% | 68.05% | 56.8% | 61.52% | 68.57% | 63.54% | 71.35% | 68.36% | 65.32% | 4.40 |
