# Supplementary material for: Revisiting sylvian fissure dissection - A preliminary investigation into surgical process modelling for evaluating surgical proficiency
Source: Brain Spine. 2025 May 21;5:104284. doi: 10.1016/j.bas.2025.104284 (PMC12171760; doi:10.1016/j.bas.2025.104284)
Supplement: Multimedia component 3 [file mmc3.docx]

| **TABLE 3 \| Surgical Duration** | **Case 1** | **Case 2** | **Case 3** | **Case 4** | **Case 5** | **Case 6** | **Case 7** | **Case 8** | **Median** | **Q1-Q3** |
| --- | --- | --- | --- | --- | --- | --- | --- | --- | --- | --- |
| **Duration intervention (hh:mm:ss)** | 21:37 | 41:02 | 17:32 | 01:58:39 | 01:06:45 | 30:04 | 24:25 | 23:58 | 27:15 | 23:23- 47:27 |
| Superficial opercular compartment | 03:58 | 03:46 | 06:50 | 36:32 | 14:25 | 06:08 | 04:23 | 06:12 | 06:10 | 04:17-08:43 |
| Deep opercular compartment | 09:59 | 05:47 | 07:31 | 27:03 | 08:46 | 04:31 | 08:06 | 07:04 | 07:49 | 06:45-09:04 |
| Cisternal Compartment | 05:49 | 01:57 | 01:00 | 13:30 | 07:51 | 01:44 | 03:60 | 02:44 | 03:22 | 01:54-06:20 |
| Aneurysm dissection | 01:51 | 29:32 | 02:11 | 41:34 | 36:03 | 17:42 | 07:56 | 07:58 | 12:50 | 06:30-31:10 |
| **Time until aneurysm dissection** | 15:38 | 11:30 | 15:21 | 1:17:10 | 30:06 | 12:22 | 16:30 | 15:60 | 15:49 | 14:37-19:54 |
