# Supplementary material for: Revisiting sylvian fissure dissection - A preliminary investigation into surgical process modelling for evaluating surgical proficiency
Source: Brain Spine. 2025 May 21;5:104284. doi: 10.1016/j.bas.2025.104284 (PMC12171760; doi:10.1016/j.bas.2025.104284)
Supplement: Multimedia component 4 [file mmc4.docx]

| **TABLE 4 \| Tool Application** | **Case 1** | **Case 2** | **Case 3** | **Case 4** | **Case 5** | **Case 6** | **Case 7** | **Case 8** | **Mean** | **Std dev.** |
| --- | --- | --- | --- | --- | --- | --- | --- | --- | --- | --- |
| **N° different tools** | 6 | 5 | 4 | 5 | 5 | 6 | 6 | 5 | 5.25 | 0.66 |
| **Tool application:** |  |  |  |  |  |  |  |  |  |  |
| Aspiration (%) | 24.00 | 42.60 | 27.60 | 41.85 | 43.60 | 27.20 | 25.10 | 39.75 | 33.96% | 8.12 |
| Microforceps (%) | 50.05 | 28.95 | 41.55 | 2.70 | 18.30 | 16.85 | 29.40 | 39.75 | 28.44% | 14.44 |
| Microscissor (%) | 1.85 | 5.35 | 14.65 | 20.40 | 16.70 | 4.35 | 8.95 | - | 10.32% | 5.93 |
| Regular forceps (%) | - | - | - | 10.10 | - | - | - | - | 10.10% | 0.00 |
| Rhoton dissector (%) | - | - | - | - | - | 5.20 | - | - | 5.20% | 0.00 |
| Ball probe (%) | - | - | - | - | 3.75 | - | - | - | 3.75% | 0.00 |
| Bipolar (%) | 0.45 | 0.35 | - | 0.25 | - | 0.55 | 4.20 | 4.90 | 1.78% | 1.97 |
| Microscalpel (%) | 0.35 | - | 0.30 | - | - | 1.30 | 1.65 | 1.20 | 0.96% | 0.54 |
| Microhook (%) | 1.00 | - | - | - | 0.10 | - | - | - | 0.55% | 0.45 |
| Water cannula (%) | - | 0.10 | - | - | - | - | 0.60 | 0.35 | 0.35% | 0.13 |
| **Retractor usage (%)** | 8.50 | 49.50 | 0.00 | 0.00 | 0.00 | 0.00 | 0.00 | 0.00 | 7.25% | 16.21 |
| **N° adverse events** | 0.28 | 0.15 | 0.23 | 0.02 | 0.01 | 0.13 | 0.20 | 0.29 | 0.16/min | 0.10 |
| Bleeding - small vessel (total) | 6 | 6 | 4 | 6 | 1 | 3 | 5 | 6 | 4.63 | 2.14 |
| Bleeding - big vessel (total) | 0 | 0 | 0 | 0 | 0 | 1 | 0 | 0 | 0.13 | 0.00 |
| Cortical lesions (total) | 0 | 0 | 0 | 0 | 0 | 0 | 0 | 1 | 0.13 | 0.00 |
| **N° Coagulations** | 0.05 | 0.05 | 0.00 | 0.00 | 0.00 | 0.07 | 0.29 | 0.33 | 0.10/min | 0.13 |
| **Mean duration coagulation (s)** | 8.04 | 5.64 | 0 | 0 | 0 | 3.06 | 6.18 | 2.87 | 2.92sec | 3.08 |
| **N° Haemostatic usage** | 0.00 | 0.00 | 0.00 | 0.02 | 0.01 | 0.03 | 0.00 | 0.00 | 0.01% | 0.01 |
| **N° microscope adjustments** | 0.37 | 0.88 | 0.86 | 1.00 | 0.87 | 0.80 | 1.02 | 1.17 | 0.87/min | 0.22 |
| Focus/zoom (total) | 0.28 | 0.54 | 0.63 | 0.84 | 0.75 | 0.50 | 0.70 | 0.92 | 0.64/min | 0.19 |
| Change of position (total) | 0.09 | 0.34 | 0.23 | 0.16 | 0.12 | 0.30 | 0.33 | 0.25 | 0.23/min | 0.09 |
| **Duration microscope adjustments (%)** | 3.30 | 5.10 | 8.50 | 10.10 | 7.70 | 7.70 | 9.10 | 5.70 | 7.15% | 2.12 |
| By assistant surgeon | 0.00 | 0.89 | 1.82 | 0.77 | 1.24 | 0.56 | 2.00 | 2.33 | 1.20% | 0.74 |
| By operating surgeon | 3.30 | 4.21 | 6.68 | 9.33 | 6.46 | 7.14 | 7.10 | 3.37 | 5.95% | 1.99 |
