# Supplementary material for: Revisiting sylvian fissure dissection - A preliminary investigation into surgical process modelling for evaluating surgical proficiency
Source: Brain Spine. 2025 May 21;5:104284. doi: 10.1016/j.bas.2025.104284 (PMC12171760; doi:10.1016/j.bas.2025.104284)
Supplement: Multimedia component 5 [file mmc5.docx]

Supplementary material

*Data pre-processing*

By default, microscope footage is automatically divided into multiple clips when exported onto an external storage. Prior to video analysis, the different fragments were merged together into one continuous video by means of the Movavi Video Converter software (Movavi Software Inc., Wildwood, USA)[23]. No additional editing in any form was performed.

The ethogram was hierarchically constituted of a *subject*, a *behaviour* and a *modifier*. Each annotation was categorized into one of two predefined activity states: either a state event or a point event. The former denotes activities or behaviours occurring over a specific timespan, marked by a starting and ending timestamp. In contrast, point events refer to actions or incidents that happen at a distinct moment in time without any temporal extension (e.g., adverse events). The annotation procedure was facilitated by the implementation of a codingmap, consisting of pre-assigned keys on the computer keyboard to assign different labels.

*Data analysis*

All surgeons were right-handed. Therefore, the number of gestures of the right hand was significantly higher and more complex as opposed to the left hand. To facilitate the analysis, the activity of the left hand was annotated separately from the right-hand actions. This was performed simultaneously with the annotation of microscope and retractor usage, as well as the occurrence of adverse events. In a second time, the labelling solely focused on the surgical actions of the right hand. In order to minimize the overall duration of the labelling procedure, the videos were examined at a rate three times faster than the standard speed.

*Result extraction*

In tabulated results obtained from the annotation software, the subject, surgical behaviour, modifier, behavioural state and time of each annotation were listed in a single row. For *state events* (e.g. tool activities), a single surgical event comprised two annotations, denoting the initiation and termination points. In other words, a surgical action was expressed in two rows. The former describing the beginning and the latter the ending of the surgical event. The duration of a surgical event could be determined by subtracting the time of the first row (start) from the second row (ending). For *point* events (e.g. adverse events), the surgical event consists of solely one observation.

Upon the conclusion of the labelling process, a tabulated summary was generated of all labelled surgical events and single observations. As such, the subject, surgical behaviour, modifier, behavioural state and time of each annotation were listed in a single row. For *state events* (e.g. tool activities), a single surgical event comprised two annotations, denoting the initiation and termination points. In other words, a surgical action was expressed in two rows. The former describing the beginning and the latter the ending of the surgical event. The duration of a surgical event could be determined by subtracting the time of the first row (start) from the second row (ending). For *point* events (e.g. adverse events), the surgical event consists of solely one observation.

A macro was written to merge the two rows of each surgical event relating to tool activities into a single row with the duration of the activity expressed as a time-range [beginning; ending]. Consequently, this approach enabled the precise calculation of the percentage of both bi-manual and single-handed tool applications. The remaining surgical parameters were retrieved through the temporal analysis of the annotated actions and events by the BORIS software.

*Clustering analysis*

First, the SPMs of the respective surgical cases were decomposed into their fundamental components, represented by three separate subject-specific index-plots. These plots delineate the sequence of actions {subject; tool; activity} undertaken by the right hand, left hand, and microscope, respectively. Subsequently, the algorithm sought the most efficient approach to align SPMs of different surgeons, known as a minimal cost path, that required the fewest alteration of the intervention structure for optimal comparison of the surgical activities displayed within the different index-plots.

The information from the right hand, left hand, and microscope was combined into a final dissimilarity (DTW) index by taking the weighted sum of the cost-paths of subject-specific index plots. The latter was iteratively computed for each pair of corresponding actions in a binary manner across the respective surgical cases (1 indicating identical surgical activities, 0 indicating otherwise). If surgeons exhibited greater differences in surgical performance or technique, substantial structural differences in the SPMS required the algorithm to make significant modifications to align the SPMs optimally, which resulted in a higher dissimilarity index. Given that all surgeons were right-handed, the right hand was deemed the most important and assigned a weight of 0.7. In turn, the left hand was given a weight of 0.2, and the microscope was assigned a weight of 0.1. Consequently, the SPMs were iteratively clustered into subsets using the average-link approach based on the degree of similarity in surgical activities within the respective cases.
